# Postoperative evolution of the addictive pattern in patients with obesity undergoing bariatric surgery

**DOI:** 10.1192/j.eurpsy.2026.10207

**Published:** 2026-07-31

**Authors:** M. Menendez Munoz, M. B. Pomar, A. S. García, C. F. Pato

**Affiliations:** 1 Psychiatry; 2 Endocrinology and Nutrition, Complejo Asistencial Universitario De León, León, Spain

## Abstract

**Introduction:**

Bariatric surgery is effective for morbid obesity, though it may be associated with mental health changes and increased substance use. This study analyzes changes in the use of addictive substances and medications before and after surgery, considering age, sex, and psychiatric history.

**Objectives:**

To assess the progression of addictive behaviors in patients undergoing bariatric surgery.

- To determine whether patients consume more abuse-prone prescription drugs (opioids, benzodiazepines, or others) before or after the intervention.

**Methods:**

A retrospective study was conducted on 126 patients who underwent surgery between 2014 and 2022. Data on demographics and the use of abuse-prone prescription drugs (such as opioids, benzodiazepines, and others) were collected before and after the intervention. Statistical analyses included chi² and McNemar tests. Measures of risk were calculated. Statistical analysis and figures were performed using Julius.ai. A p-value < 0.05 was considered statistically significant.

**Results:**

Before surgery, 62.7% of patients (79) did not use prescription drugs with abuse potential. Benzodiazepine use was 14.29% (18), opioids 7.14% (9), and combined use 7.14% (9). Data were missing for 11 patients. After surgery, only 44.44% (56) remained non-users. Benzodiazepine use decreased to 4.76% (6), opioid use rose to 33.33% (42), and combined use increased to 12.7% (16). Data were missing for 6 patients. In 114 valid pre-post pairs, the shift from non-use to use was significant (p = 0.000388), with 43.04% of pre-op non-users starting use post-op. Overall substance use rose from 30.7% to 51.75% (Figure 1). The NNH was 4.75; RR was 1.68; OR for initiation vs cessation was ~3.40.

By drug type:

Benzodiazepines dropped from 14.91% to 4.39% (70.59% reduction, p = 0.007).

Opioids rose from 7.89% to 33.33% (322.22% increase, p < 0.00001).

Combined use increased from 7.89% to 14.04% (not significant, p = 0.18).

In summary, opioid use increased significantly post-surgery while benzodiazepine use declined (Figure 2).

**Image 1:**
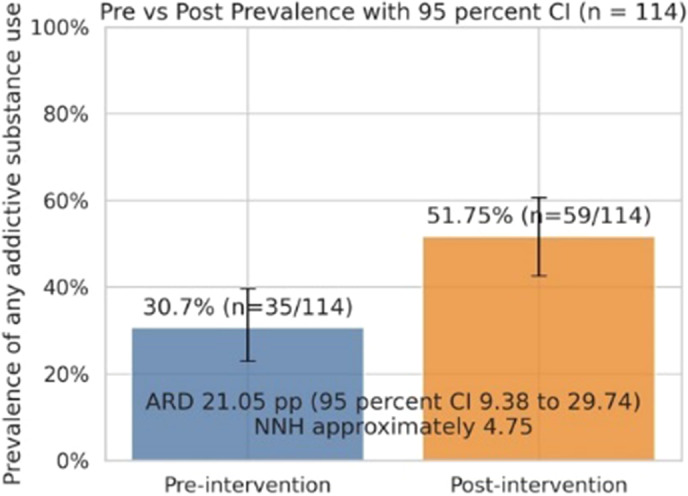
Image 1: Long description.

**Image 2:**
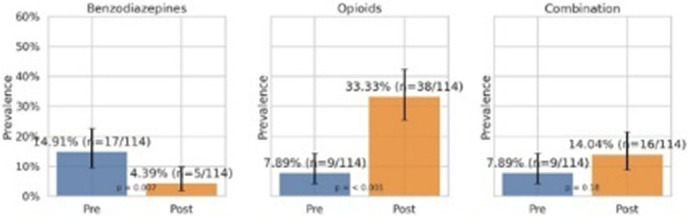
Image 2: Long description.

**Conclusions:**

Based on the data presented, it appears that patients exhibit an addictive phenotype that migrates from food to other substances. This highlights the importance of addressing addiction not merely in its individual forms, but as a broader behavioral pattern. Given that many addictions share a common neurobiological pathway—particularly involving reward, impulse control, and reinforcement mechanisms—it is essential to focus interventions on the underlying addictive process itself. A comprehensive, integrative approach to addiction may help prevent the substitution of one pathological behavior for another.

**Disclosure of Interest:**

None Declared

## Long descriptions

### Image 1: Long description

A bar chart titled Pre vs Post Prevalence with 95 percent C I (n = 114). The vertical Y-axis is labeled Prevalence of any addictive substance use, ranging from 0% to 100% in 20% increments. The horizontal X-axis has two categories.

* Pre-intervention: A blue bar reaching 30.7% (n = 35/114) with a black error bar extending from approximately 23% to 40%.

* Post-intervention: An orange bar reaching 51.75% (n = 59/114) with a black error bar extending from approximately 43% to 61%.

Centered at the bottom of the chart area is a text block stating: A R D 21.05 p p (95 percent C I 9.38 to 29.74) N N H approximately 4.75.

Navigate back to Image 1:.

### Image 2: Long description

A three-panel bar chart set. Each chart shares a Y-axis labeled Prevalence ranging from 0 percent to 60 percent and an X-axis with two categories: Pre (blue bar) and Post (orange bar). All bars include error bars and sample size notations (n over 114).

* Left Panel (Benzodiazepines): The Pre bar is at 14.91 percent (n = 17/114). The Post bar decreases to 4.39 percent (n = 5/114). A p-value of 0.007 is noted between the bars.

* Middle Panel (Opioids): The Pre bar is at 7.89 percent (n = 9/114). The Post bar increases significantly to 33.33 percent (n = 38/114). A p-value of less than 0.001 is noted.

* Right Panel (Combination): The Pre bar is at 7.89 percent (n = 9/114). The Post bar increases to 14.04 percent (n = 16/114). A p-value of 0.18 is noted.

Navigate back to Image 2:.

